# Environmental temperatures significantly change the impact of insecticides measured using WHOPES protocols

**DOI:** 10.1186/1475-2875-13-350

**Published:** 2014-09-03

**Authors:** Katey D Glunt, Krijn P Paaijmans, Andrew F Read, Matthew B Thomas

**Affiliations:** Center for Infectious Disease Dynamics, The Pennsylvania State University, University Park, Pennsylvania, PA USA; Department of Biology, The Pennsylvania State University, University Park, Pennsylvania, PA USA; Department of Entomology, The Pennsylvania State University, University Park, Pennsylvania, USA; Barcelona Centre for International Health Research, (CRESIB, Hospital Clínic-Universitat de Barcelona), Barcelona, Spain

**Keywords:** *Anopheles stephensi*, Insecticide, Malaria vectors, Mosquito control, Temperature

## Abstract

**Background:**

Insecticides are critical components of malaria control programmes. In a variety of insect species, temperature plays a fundamental role in determining the outcome of insecticide exposure. However, surprisingly little is known about how temperature affects the efficacy of chemical interventions against malaria vectors.

**Methods:**

*Anopheles stephensi,* with no recent history of insecticide exposure, were exposed to the organophosphate malathion or the pyrethroid permethrin at 12, 18, 22, or 26°C, using the WHO tube resistance-monitoring assay. To evaluate the effect of pre-exposure temperature on susceptibility, adult mosquitoes were kept at 18 or 26°C until just before exposure, and then moved to the opposite temperature. Twenty-four hours after exposure, mosquitoes exposed at <26°C were moved to 26°C and recovery was observed. Susceptibility was assessed in terms of survival 24 hours after exposure; data were analysed as generalized linear models using a binomial error distribution and logit link function.

**Results:**

Lowering the exposure temperature from the laboratory standard 26°C can strongly reduce the susceptibility of female *An. stephensi* to the WHO resistance-discriminating concentration of malathion (*χ*^2^_df=3_ = 29.0, p < 0.001). While the susceptibility of these mosquitoes to the resistance-discriminating concentration of permethrin was not as strongly temperature-dependent, recovery was observed in mosquitoes moved from 12, 18 or 22°C to 26°C 24 hours after exposure. For permethrin especially, the thermal history of the mosquito was important in determining the ultimate outcome of insecticide exposure for survival (permethrin: *pre-exposure temperature*: F_1,29_ = 14.2, p < 0.001; *exposure temp*: F_1,29_ = 1.1, p = 0.3; *concentration*: F_1,29_ = 85.2, p < 0.001; *exposure temp* x *conc*: F_1,29_ = 5.8, p = 0.02). The effect of acclimation temperature on malathion susceptibility depended on the exposure temperature (*exposure temp*: F_1,79_ = 98.4, p < 0.001; *pre-exposure temp*: F_1,79_ = 0.03, p = 0.9; *pre-exp temp* x *exp temp* F_1,79_ = 6.0, p = 0.02).

**Conclusions:**

A single population of *An. stephensi* could be classified by WHO criteria as susceptible or resistant to a given chemical, depending on the temperature at which the mosquitoes were exposed. Investigating the performance of vector control tools under different temperature conditions will augment the ability to better understand the epidemiological significance of insecticide resistance and select the most effective products for a given environment.

## Background

Chemical insecticides form the backbone of malaria vector control programmes. Deployed on insecticide-treated nets (ITNs), long-lasting, insecticide-treated nets (LLINs), or as indoor residual sprays (IRS), these compounds aim to incapacitate or kill adult mosquitoes on contact. The consequent reduction in the density and average lifespan of vector mosquitoes effectively reduces transmission and, hence, disease incidence [1]. However, the effectiveness of chemical insecticides is now being threatened by the widescale emergence of insecticide resistance [2–4].

**Figure 1:**
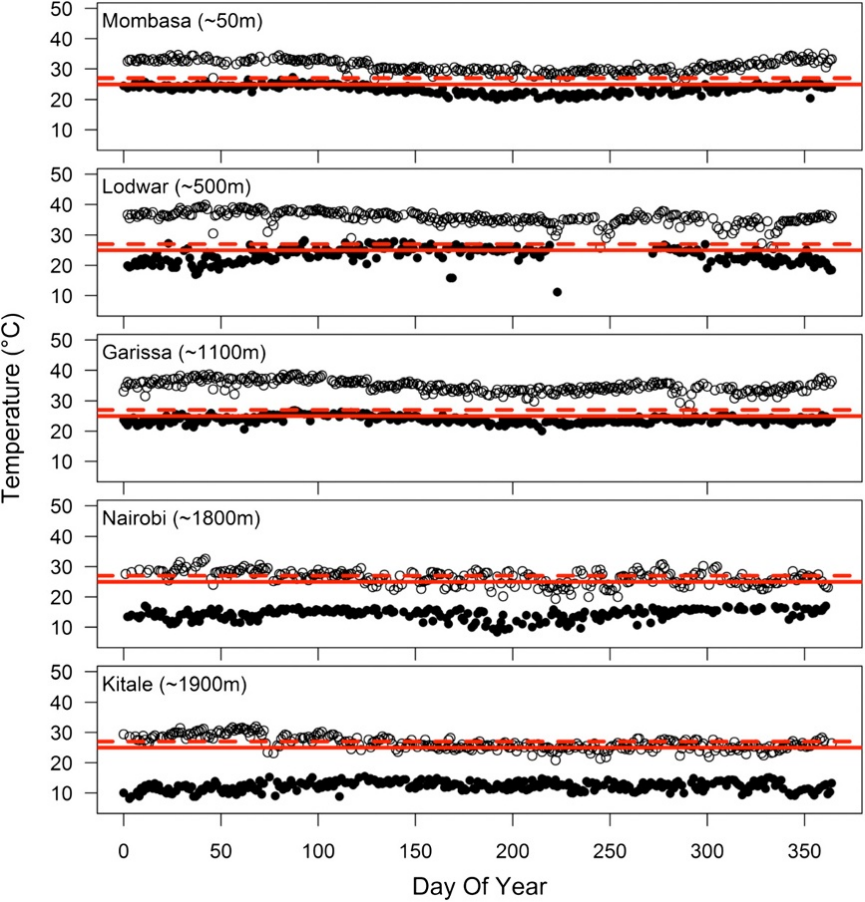
**Annual temperature profiles at five meteorological-stations in Kenya, 2011.** Solid circles indicate daily minimum temperatures and open circles indicate daily maximum temperatures. These locations, arranged top-to-bottom from lowest to highest altitude, are presented regardless of malaria transmission levels (Mombasa [9], Lodwar [10], Garissa [11], Nairobi [12], Kitale [13]), as a sample of the diversity of local temperature conditions. The WHO recommends that insecticide resistance testing be conducted at 25°C [6], indicated by the solid red line (previously, 27°C [5]; dashed line). Data obtained from NOAA, http://www.ncdc.noaa.gov/cdo-web.

The standard methodologies for monitoring and evaluating resistance use bioassay protocols developed by the WHO Pesticide Evaluation Scheme (WHOPES) to test the mortality of young (three- to five-day old) female mosquitoes following single, limited-time exposure to the relevant insecticide [5, 6]. For many years the recommended test temperature was 27 ± 2°C [5], although this has recently been lowered to 25 ± 2°C [6]. However, temperatures in the field can vary considerably, especially during the night when mosquitoes are actively searching for hosts ([7, 8] and see Figure 1]). Determining the impact of current and future insecticides under realistic environmental conditions could be important for making informed decisions about which compound is likely to be effective under local epidemiological conditions [8].

Studies on a diversity of insects have shown the toxicity of different classes of chemical insecticide to be strongly influenced by temperature, even within temperature ranges relevant to the functionality of the compounds in the field ([14, 15] cited in [16]). For example, in *Diaphorina citri* (Hemiptera: Psyllidae), a pest and vector of greening disease in citrus, Boina *et al.* saw that two organophosphates and a carbamate were more toxic at higher temperatures, while three out of four of the tested pyrethroid insecticides (zeta-cypermethrin, fenpropathrin, lambda-cyhalothrin) were more toxic at lower temperatures [17]. Bifenthrin, the fourth pyrethroid tested, was more toxic at higher temperatures [17], which shows that even within the same class of chemical, against the same species of insect, the interaction between the toxicity of a given compound and temperature can be difficult to predict *a priori* (see also in tobacco budworm [18]).

As in other insects, nervous system sensititivity [19] and metabolic activity [20] are highly temperature-dependent in mosquitoes. Given that the currently available public health insecticides are neurotoxins and that metabolic detoxification plays an important role in insecticide resistance, it is likely that the efficacy of insecticides against mosquito vectors will exhibit some temperature dependence. While not extensively researched, at least two studies support this assertion. Hodjati and Curtis [16] exposed *Anopheles stephensi* to the pyrethroid permethrin and observed that fewer mosquitoes died at 22 than at 16°C, but mortality following exposure increased as the temperature increased from 22 to 37°C. *Anopheles gambiae*, on the other hand, displayed a positive temperature coefficient, or relationship between temperature and chemical response, with mortality increasing as temperature increased [16]. In another study using *An. stephensi* [21], the organophosphate diazinon killed more mosquitoes at higher temperatures, while fewer mosquitoes died following DDT exposure as the temperature increased from 20 to 30°C. These studies indicate that mosquito species, chemical compound and local temperature conditions can all contribute to the efficacy of an insecticidal intervention.

The current study investigates how temperature affects knockdown and mortality of *An. stephensi* females following exposure to two classes of insecticide approved for use in public health: organophosphates (malathion) and pyrethroids (permethrin) [22]. A long-standing laboratory strain of mosquito with no recent history of insecticide exposure was exposed to WHO-prescribed resistance-discriminating insecticide concentrations. Mosquitoes were also exposed to lower insecticide doses, simulating exposure to decaying spray residues (or possibly very transient contact), as must happen in the field [23, 24]. Exposures were conducted across a range of temperatures, including standard WHO conditions and a series of cooler temperatures likely representative of night time conditions when mosquitoes are actively host searching. The results indicate that the single population of *An. stephensi* could be classified by WHO criteria as susceptible or resistant to a given chemical, depending on the temperature at which the mosquitoes were housed.

## Methods

### Experimental overview

**Figure 2:**
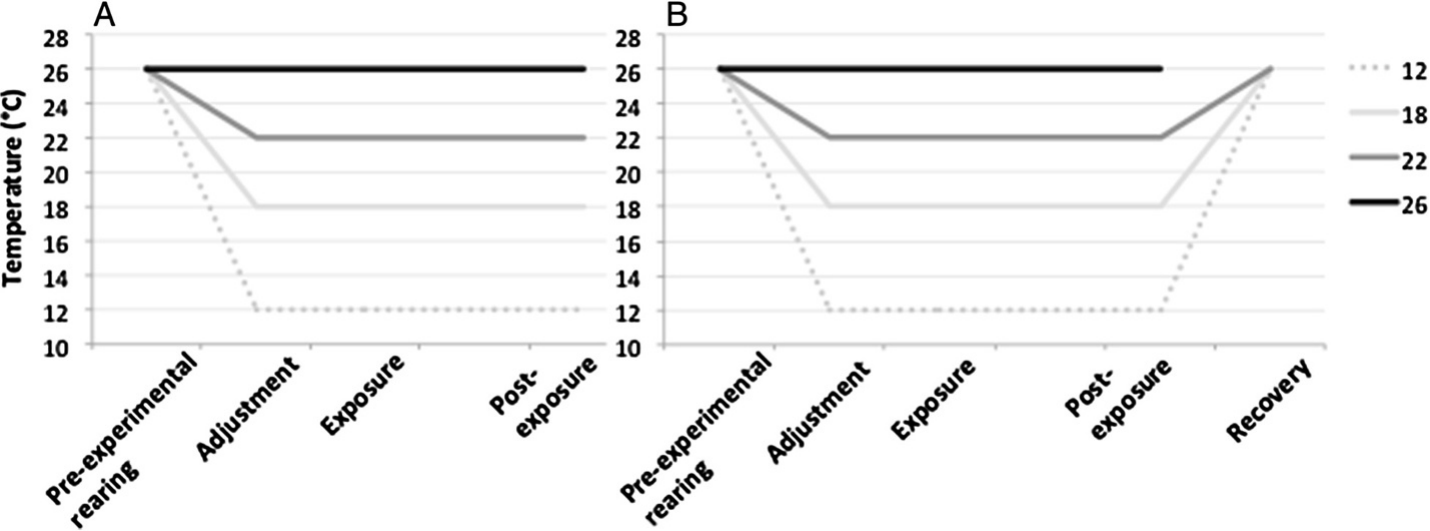
**Temperature history profiles of mosquitoes exposed to A) malathion or B) permethrin.** All adults were reared under standard 26°C conditions. Up to one hour prior to exposure, females in each treatment group were moved to their exposure temperatures to adjust to the holding tubes; they remained at these temperatures throughout and after their hour-long insecticide exposure. After 24 hours at their treatment temperature, mosquitoes exposed to permethrin were moved to 26°C for 15 minutes in order to assess recovery.

**Figure 3:**
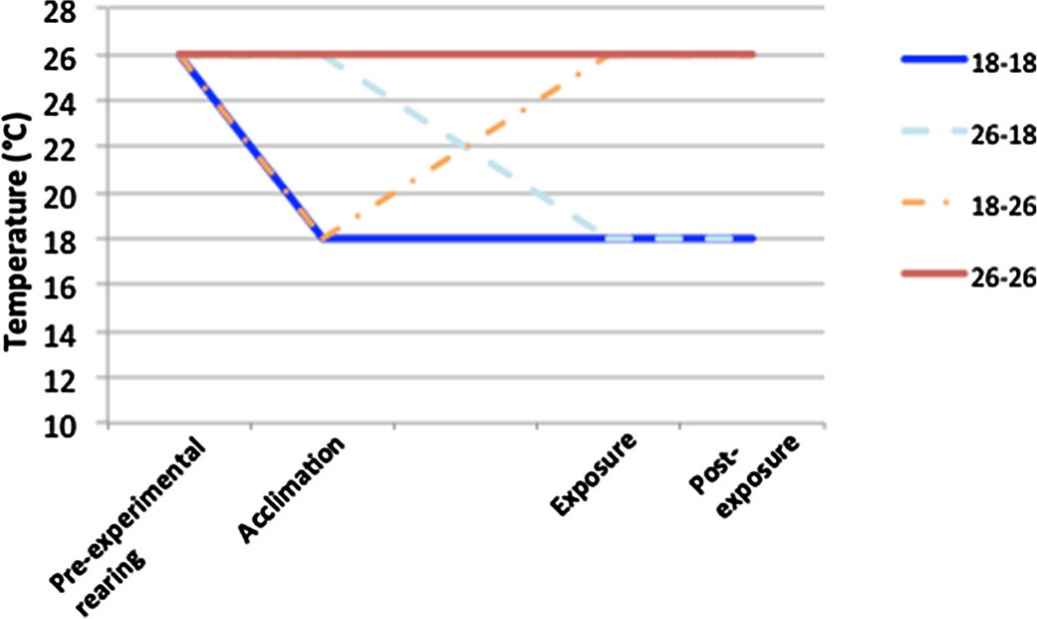
**Temperature history profiles of mosquitoes allowed to acclimate to different temperatures before insecticide exposure.** Larvae were reared under standard 26°C conditions. Within two days of pupation, a cohort of mosquitoes was divided in half and moved to cages at 18 or 26°C to acclimate to those temperatures for up to five days. Each half of the cohort was divided prior the insecticide exposure, to create four treatment groups based on acclimation and exposure temperatures. After being separated into WHO tubes, mosquitoes remained at exposure temperatures throughout and for 24 hours after their hour-long insecticide exposure. After 24 hours at their treatment temperature, mosquitoes exposed to permethrin were moved to 26°C for 15 minutes in order to assess recovery.

Two types of experiments were conducted, with all insecticide exposures following the standard insecticide-resistance monitoring ‘tube test’ protocol from the WHO [22], except for the temperature(s) at which mosquitoes were exposed. First, to evaluate the effect of exposure temperature on insecticide susceptibility (referred to as ‘Exposure temperature experiments’), mosquitoes were exposed to the resistance-discriminating dose of malathion or permethrin at 12, 18, 22, or 26°C (Figure 2). The lower temperatures repesent cooler night time temperatures, whereas 26°C is intermidiate to the previous (27°C) and current (25°C) WHO-prescribed temperatures. This approach takes mosquitoes from standard insectary conditions (26°C) and transfers them instantaneously into the different test temperatures. In order to assess whether this rapid change in temperature affected the outcome of insecticide exposure, a second set of assays were conducted in which mosquitoes were given a period of acclimation at either 18 or 26°C for at least three days prior to subsequent exposure at either 18 or 26°C (Figure 3, ‘Acclimation experiments’).

### Insecticide-treated paper preparation

Filter paper sheets were impregnated with technical-grade insecticide (ChemService, West Chester, PA, USA) or control solution, according to WHO protocol [22], at least 24 hours prior to use. Acetone acted as the solvent; olive oil (Sigma-Aldrich, St. Louis, MO, USA) served as the carrier for malathion and silicon oil (Dow Chemical, Midland, MI, USA) for permethrin (57.3:42.1 *cis:trans*). Concentrations were calculated based on the mg of active ingredient per unit of oil [22]. The WHO resistance-discriminating concentrations are 5% malathion and 0.75% permethrin; in the acclimation experiments, which also used lower concentrations, the concentrations were selected based on previous work in this laboratory [25, 26]. The discriminating concentrations were used in order to examine how the outcome of resistance monitoring tests changes under different conditions. The lower concentrations were used to explore what happens when not all mosquitoes are killed by exposure, as might occur when spray residues have decayed, when there is only very transient contact, or possibly when mosquitoes exhibit some resistance (though whether the effect of a reduced dose is directly analogous to partial resistance is not clear).

### Exposure temperature experiments

*Anopheles stephensi* with no recent exposure to insecticides were obtained from the US National Institutes of Health and cultured at The Pennsylvania State University since 2008 under standard insectary conditions of 26 ± 1°C and 80 ± 5% relative humidity as described in Glunt *et al*. [25].

For each insecticide exposure, approximately 25 female mosquitoes per replicate were transferred by mouth aspirator to plain paper-lined holding tubes. Tubes were moved to their respective temperature treatments (12, 18, 22°C) or kept at 26°C, and mosquitoes were given between 30 minutes and one hour to acclimate. Mosquitoes were then transferred into the connecting insecticide-lined tubes and exposed for one hour. At the end of the one-hour exposure period, knockdown was scored (see below), and mosquitoes were gently blown into mesh-covered paper cups and provided with glucose. For each experiment, mosquitoes were exposed in four consecutive experimental blocks during a single day, with each treatment group represented in each block, so that there were four replicates for each treatment. Malathion experiments were additionally replicated in time. Therefore, the total number of mosquitoes exposed to insecticide at each temperature was around 100 for permethrin, and 200 for malathion (exact sample sizes in Table 1). Post-exposure, mosquitoes were kept at their respective exposure temperatures for 24 hours and then mortality was recorded.

**Table 1:** **Number of mosquitoes exposed to each temperature treatment in exposure temperature experiments**

	Control	Permethrin	Control 1	Malathion 1	Control 2	Malathion 2
**12°**	94	95	82	98	96	98
**18°**	91	103	98	101	99	101
**22°**	99	98	89	98	99	97
**26°**	97	98	100	93	100	98

In accordance with WHO protocols, each exposure temperature experiment included four replicates of approximately 25 females for the treatment groups exposed to the resistance-disciminating concentrations (permethrin: 0.75%; malathion: 5%). Groups of control mosquitoes, exposed to insecticide-free papers, were likewise replicated. There were two experiments in which mosquitoes were exposed to malathion.

The symptoms of pyrethroid exposure have been shown to be quickly reversible through manipulations of ambient temperature (unless of course the insects are genuinely dead) [15]. To examine this effect, after recording knockdown/mortality 24 hours post-exposure, all cups with mosquitoes that were exposed to permethrin at 12, 18 and 22°C were moved to 26°C. Mosquitoes at 26°C were all dead. The number of mosquitoes able to fly when air was blown into the cup and/or the cup was tapped after 15 minutes at the new temperature was then counted (Figure 2B, ‘Recovery’). Malathion exposure does not generate a knockdown phenotype, and so recovery was not observed for this chemical.

### Acclimation experiments

To determine if a sudden change in temperature, rather than the temperature itself, could have affected how mosquitoes responded to insecticide exposure,a cohort of adults reared under standard insectary conditions (26°C) was split and placed at either 18° ± 0.5°C or 26° ± 0.5°C at around the time of emergence. Adult mosquitoes experienced the acclimation temperatures between three and five days prior to insecticide exposure (Figure 3, ‘Acclimation’).

Except for the acclimation period, insecticide exposures followed the WHO insecticide-resistance assay protocol [5] as explained for the exposure temperature experiments. Half of the mosquitoes were exposed to insecticides in the temperature to which they were acclimated, and half were exposed at the other temperature. Therefore, there were four treatment groups, distinguished according to their acclimation and exposure temperatures (acclimation T°C-exposure T°C): 18–18, 18–26, 26–18, and 26–26. In addition to the control (0%) and discriminating concentrations of each chemical (malathion: 5%; permethrin: 0.75%), females were exposed to one or more concentrations expected to give intermediate levels of mortality (malathion: 0.25, 0.5, 0.75%; permethrin: 0.25%). There were four replicates of ~25 mosquitoes for each treatment group and control/chemical concentration (exact sample sizes given in Table 2). Recovery was also assessed in mosquitoes exposed to 0.75% permethrin at 18°C (18–18 and 26–18 groups).

**Table 2:** **Number of mosquitoes exposed to each temperature treatment in acclimation experiments**

	Permethrin [%]			Malathion [%]				
	0	0.25	0.75	0	0.25	0.5	0.75	5.0
**18-18**	112	103	103	92	98	92	93	94
**26-18**	99	99	106	186	179	182	88	95
**18-26**	103	75	79	192	93	93	93	101
**26-26**	103	103	102	183	190	184	94	100

Each temperature treatment and insecticide concentration were typically replicated four times, approximately 25 mosquitoes per replicate. In the permethrin experiment, mosquitoes escaped and replicates were dropped in two cases (group 18–26, 0.25 and 0.75%). Some treatment groups exposed to malathion or malathion controls were additionally replicated in time (26–18 and 26–26: 0, 0.25 and 0.5%; 18–26: 0%).

### Data analysis

At the end of the one-hour exposure period, before transferring the mosquitoes to cups, the exposure tube was gently tapped and rotated to initiate flight in any mosquitoes prone but able to fly. Mosquitoes that did not fly were scored as dead; from these counts, one hour survival was calculated. One day after each exposure, we counted the number of dead mosquitoes in each cup and calculated 24-hour survival. In experiments using permethrin, after 24-hour mortality was recorded, mosquitoes from the lower-temperature treatment groups that were able to fly at the end of a 15-minute period at 26°C were counted to calculate recovery.

Statistical analyses were performed in SPSS v.20 (PASW 20.0) and R v. 2.10.1 [27]. When possible, data on one hour and 24 hours survival from exposure temperature and acclimation experiments were analysed as generalized linear models using a binomial error distribution and logit link function. Quasibinomial distribution and logit link were used in cases of overdispersion. In models with more than one independent variable, the maximal model was fitted with the interaction terms first and non-significant interactions were removed by backward-elimination. In the exposure temperature experiments, exposure temperature (12, 18, 22, 26°C) was the only independent variable. In the acclimation temperature experiments, concentration was included as an independent variable (malathion: 0.25, 0.50, 0.75, 5%; permethrin: 0.25, 0.75%), along with acclimation and exposure temperature (both either 18 or 26°C).

### Resistance classification

‘Resistance’ to a given chemical was designated according to the current WHO criteria [6], based on the level of mortality observed 24 hours after insecticide exposure. Mosquito populations are classified as resistant if, after two resistance tests, more than 2% of the exposed individuals survive. When multiple tests cannot be carried out, mortality less than 98% indicates that further investigation is needed to determine resistance status. This level was recently changed from mortality less than 80% [5].

## Results

### Exposure and acclimation temperature experiments: malathion

**Figure 4:**
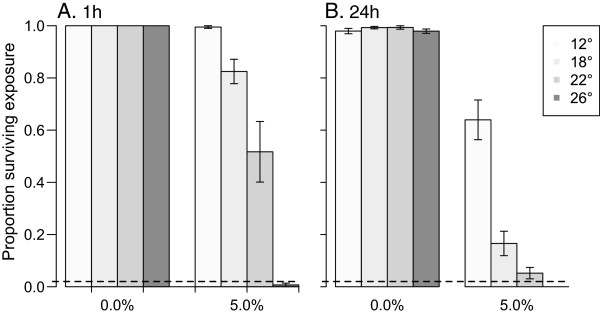
**Effect of exposure temperature on susceptibility to malathion.** Panels depict survival of female *An. stephensi* **A)** one hour and **B)** 24 hours after exposure to 0 or 5% malathion at different temperatures. The dashed line at 0.02 indicates the level of survival above which populations are classified as resistant by the WHO [6].

Exposure temperature strongly influenced the outcome of exposure of female *An. stephensi* to the WHO resistance-discriminating dose of malathion. At 5%, this organophosphate displayed a positive temperature coefficient of toxicity between 12 and 26°C, with mortality one hour and 24 hours after exposure increasing with exposure temperature (Figure 4A, one hour, *Temperature*: *χ*^2^_df=3_ = 66.2, p < 0.001; 4b) 24 hours, *Temp*: *χ*^2^_df=3_ = 29.0, p < 0.001). The groups of females exposed at temperatures less than 26°C would be classified as malathion resistant by current WHO criteria [6].

**Figure 5:**
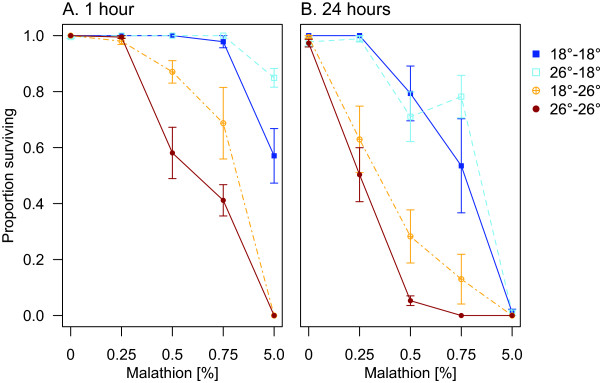
**Effect of temperature history on susceptibility to malathion.** Panels depict survival of female *An. stephensi* **A)** one hour and **B)** 24 hours after exposure. Error bars (±1 SE) are centred at the mean survival of each treatment group, at each concentration of malathion. To show the relationship between dose and temperature treatment, lines connect the responses of each treatment group across increasing concentrations.

The results of the acclimation experiments were consistent with the exposure temperature experiments: exposure to malathion at lower temperatures was associated with lower mortality. At one hour after exposure, acclimation temperature affected females’ susceptibility to the compound, but its effect depended on malathion concentration (Figure 5A and Table 3; *Acclimation temp*: F_1,79_ = 0.5, p = 0.5; *Exposure temp*: F_1,79_ = 98.2, p < 0.001; *Concentration*: F_3,79_ = 81.1, p < 0.001;0 *Accl temp* × *Conc* F_3,79_ = 4.5, p = 0.01). After 24 hours, exposure temperature and malathion concentration were still important in explaining the proportion of females that died (Figure 5B and Table 3; *Exposure temp*: F_1,79_ = 98.4, p < 0.001; *Concentration*: F_3,79_ = 81.5, p < 0.001). The effect of acclimation temperature depended on the exposure temperature (*Acclimation temp*: F_1,79_ = 0.03, p = 0.9; *Accl temp* × *Exposure temp* F_1,79_ = 6.0, p = 0.02).

**Table 3:** **Effect of acclimation temperature on insecticide susceptibility**

	Malathion: 1 h			Malathion: 24 h			Permethrin		
	df	F	p-value	df	F	p-value	df	F	p-value
Acclimation temperature	1	0.5	0.5	1	0.03	0.9	1	14.2	**0.001**
Exposure temperature	1	98.2	**<0.001**	1	98.4	**<0.001**	1	1.1	0.3
Concentration	3	81.1	**<0.001**	3	81.5	**<0.001**	1	85.2	**<0.001**
Acclimation temp. x Exposure temp.	1	3.6	0.06	1	6.0	**0.02**	1	3.0	0.1
Acclimation temp. x Concentration	3	4.5	**0.01**	3	1.8	0.2	1	0.1	0.7
Exposure temp. x Concentration	3	1.3	0.3	3	0.5	0.7	1	5.8	**0.02**
Accl. temp. x Exp. temp. x Concentration	3	0	1.0	3	1.3	0.3	1	1.0	0.3

Generalized linear models results from temperature-acclimation experiments. Significance was evaluated at α = 0.05, and significant terms are indicated by boldface type. Survival at one hour after malathion exposure depended on the exposure temperature; the influence of acclimation temperature on one-hour survival depended on malathion concentration. At 24 hours post-malathion-exposure, survival depended on both acclimation and exposure temperature, but acclimation temperature was only important at certain exposure temperatures. Acclimation temperature significantly influenced permethrin survival independent of chemical concentration, while the effect of exposure temperature on survival depended on concentration.

### Exposure and acclimation temperature experiments: permethrin

**Figure 6:**
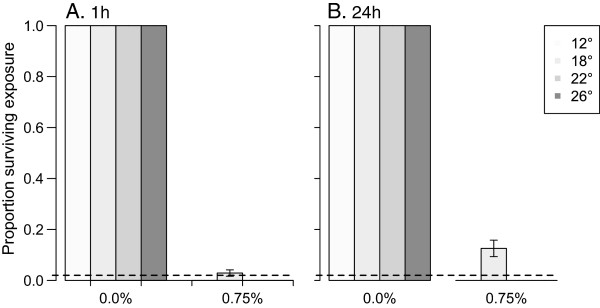
**Effect of exposure temperature on susceptibility to permethrin.** Panels depict survival of female *An. stephensi* **A)** one hour and **B)** 24 hours after exposure to 0 or 0.75% permethrin at different temperatures (Mean +/-1SE). The dashed line at 0.02 indicates the level of survival above which populations are classified as resistant by the WHO [6].

Ambient temperature during the exposure of female *An. stephensi* to 0.75% permethrin had little impact on their susceptibility, according to the standard WHO resistance-evaluation criteria (Figure 6) [6]. At the end of the one-hour exposure, no control females were affected, but all permethrin-exposed females were knocked down. After 24 hours at treatment temperatures, all control females were alive, whereas no females survived permethrin exposure at 12, 22, or 26°C. At 18°C, 13% (SEM = 0.05%) of females survived (note, although internally replicated, this experiment was not repeated through time, so it is possible that this increased survival at one temperature is an artefact; however, enhanced survival at the intermediate temperature is consistent with results of the previous study of Hodjati and Curtis [16]).

**Figure 7:**
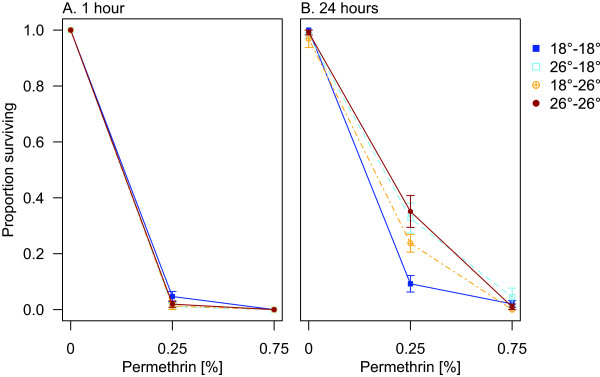
**Effect of temperature history on susceptibility to permethrin.** Panels depict survival of female *An. stephensi* **A)** one hour and **B)** 24 hours after exposure. Error bars (+/-1SE) are centred at the mean survival of each treatment group, at each concentration. Lines connect separate groups of mosquitoes, exposed to increasing concentrations of permethrin, to show the relationship between dose and temperature treatment.

When females were exposed to a lower concentration of permethrin, acclimation and exposure temperature were both important in determining the survival outcome 24 hours after exposure (Figure 7 and Table 3; 24 hours, *Acclimation temperature*: F_1,29_ = 14.2, p < 0.001; *Exposure temp*: F_1,29_ = 1.1, p = 0.3; *Concentration*: F_1,29_ = 85.2, p < 0.001; *Exposure temp* × *Conc*: F_1,29_ = 5.8, p = 0.02). For group 26–18, in which the acclimation temperature was warmer than the exposure temperature, mosquitoes were more likely to survive exposure than the group that was constantly at 18°C (18–18). When acclimated to a temperature cooler than the exposure temperature (18–26), mosquito survival was lower than in those constantly at 26°C (26–26). At one hour after exposure, none of the groups differed in their knockdown rates, at either concentration.

**Figure 8:**
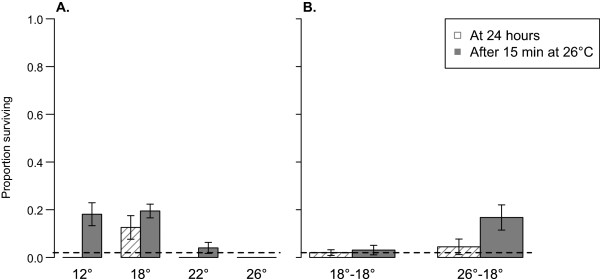
**Recovery of females exposed to permethrin at temperatures lower than 26°C.** Hatched bars reflect survival 24 hours after exposure to permethrin at **A)** 12, 18 or 22°C in exposure temperature experiments or **B)** 18°C in acclimation experiments, using scoring criteria described by the WHO resistance-monitoring assay. Solid bars show the proportion of females surviving by this same measure after those groups experienced 15 minutes at 26°C. The recovery of these females suggests that post-exposure temperature can influence insecticide susceptibility. The dashed line at 0.02 indicates the level of survival above which populations are classified as ‘resistant’ by the WHO.

Although they were scored as ‘dead’ because they were unable to fly at 24 hours post-exposure, many of the prone females that had been exposed to permethrin at temperatures lower than 26°C were still moving their legs. Therefore, they were moved to 26°C to check for recovery. After just 15 minutes at 26°C, a number of mosquitoes were able to fly. The final proportion of survival in these groups of mosquitoes would allow both to be classified as resistant according to WHO criteria (Figure 8A) [6]. Though recovery was not substantial in the acclimation experiment mosquitoes that had been acclimated to and exposed at 18°C (18–18), the mosquitoes that had been reared at the typical 26°C and exposed at 18°C recovered enough to then be classified as resistant (Figure 8B).

## Discussion

Temperatures before, during and after exposure can influence how well the organophosphate, malathion, and the pyrethroid, permethrin, kill mosquitoes. Here, a single population of *An. stephensi* could be classified by WHO criteria as either susceptible or resistant to a given chemical, depending on the temperature at which the mosquitoes were exposed. The exposure temperatures investigated in the current study were selected to be lower than the standard WHO test temperatures to simulate contact during the evening and nighttime hours when ambient temperatures tend to be cooler ([8], and see Figure 1]). However, conditions can also be much hotter, particularly in indoor environments during the day [28], and these temperatures might also have an effect.

Some of the effects of temperature observed were only apparent with exposures to concentrations of chemical much lower than the resistance-discriminating concentrations. Though insecticide application rates in the field are set deliberately high to accommodate variation in conditions or mosquito susceptibility, exposure of mosquitoes to lower concentrations would be expected as products decay over time [23, 29], or where contact with a treated susbtrate is only transient, which might be especially common in the case of the excito-repellent pyrethroids [7, 30]. Intentionally exposing mosquitoes to reduced insecticide concentrations, or to insecticides for shorter durations, could reveal potentially subtle but important effects of intrinsic factors like insect age or condition [25], or extrinsic factors like temperature, on insecticide susceptibility.

Moreover, while the current study used a nominally susceptible laboratory strain, it is possible that the effects of temperature during and post exposure could be even greater for mosquitoes expressing some level of physiological resistance (analgous to the ‘dose × temperature’ interactions observed using lower doses on this susceptible strain). This suggestion is supported by recent research demonstrating that variation in temperature and time of day can alter diverse aspects of mosquito physiology and immunity [31, 32]. How expression of insecticide resistance in malaria vectors varies across realistic variation in environmental conditions remains an open question.

The current study also reveals complexities in interpreting the different end points used in the WHO assays. While all females were scored as dead 24 hours after exposure to permethrin at 12, 18 or 22°C (i.e., they would be classified as fully susceptible to permethrin), a small proportion recovered when they were moved to 26°C (Figure 8A). A similar effect was observed in the acclimation treatments (8B). According to WHO criteria, the ultimate level of survival would justify additional testing to evaluate this ‘potentially-resistant’ population [6]. Post-exposure temperatures have been shown to decrease or increase insecticidal activity in other insects [14–17, 33–36], but this appears not to have been investigated extensively with malaria vectors. More generally, the functional significance of ‘knockdown’ remains unclear. A common expectation is that if mosquitoes are knocked down they will be eaten by predators or possibly trodden on or swept away during regular cleaning. If this is the case, then potential for recovery is likely irrelevant, as knocked down insects are functionally dead. However, if this is not the case, the extent to which temperature variation affects the ability of mosquitoes to recover following exposure could be an important factor for understanding impact of insecticides (including resistance) in different transmission settings.

Determining the epidemiological significance of insecticide resistance requires a thorough understanding of how insecticides work (or fail to work) under field conditions. Unfortunately, the relationship between the outputs of lab-based resistance-monitoring tests and impacts on ultimate control is complex [37]. While the current study revealed effects of temperature, the mechanisms involved were not studied explicitly. Temperature could affect dose-transfer and acquisition of a chemical through effects on mosquito activity. Additionally, temperature could impact rates of cuticle penetration, insecticide/target-site-interactions or insecticide detoxification enzyme activity. Regardless, the current results, together with those of Hadaway and Barlow [21] and Hodjati and Curtis [16], support the suggestion that insecticide-testing protocols could benefit from the addition of temperatures other than the standard 25 ± 2°C [8]. Inclusion of temperatures representing the seasonal minima and maxima, for example, could provide a useful indication of how variation in local conditions could affect insecticide efficacy and the expression of resistance.

## Abbreviations

IRS: Indoor residual spraying
ITNs: Insecticide-treated nets
LLINs: Long-lasting insecticide-treated nets
WHO: World Health Organization
WHOPES: World Health Organization Pesticide Evaluation Scheme.

## References

[1] WHO (2012). World Malaria Report 2012. pp. 293.

[2] Maxmen A (2012). Malaria surge feared. Nature.

[3] Kelly-Hope L, Ranson H, Hemingway J (2008). Lessons from the past: managing insecticide resistance in malaria control and eradication programmes. Lancet Infect Dis.

[4] Ranson H, N'Guessan R, Lines J, Moiroux N, Nkuni Z, Corbel V (2011). Pyrethroid resistance in African anopheline mosquitoes: what are the implications for malaria control?. Trends Parasitol.

[5] WHO (1998). Test procedures for insecticide resistance monitoring in malaria vectors, bio-efficacy and persistence of insecticide on treated surfaces.

[6] WHO (2013). Test procedures for insecticide resistance monitoring in malaria vector mosquitoes pp. 39.

[7] Pates H, Curtis C (2005). Mosquito behavior and vector control. Annu Rev Entomol.

[8] Glunt KD, Blanford JI, Paaijmans KP (2013). Chemicals, climate, and control: increasing the effectiveness of malaria vector control tools by considering relevant temperatures. PLoS Pathog.

[9] Pond B (2013). Malaria indicator surveys demonstrate a markedly lower prevalence of malaria in large cities of sub-Saharan Africa. Malar J.

[10] Bayoh MN, Akhwale W, Ombok M, Sang D, Engoki S, Koros D, Walker E, Williams H, Burke H, Armstrong G, Cetron MS, Weinberg M, Breiman R, Hamel MJ (2011). Malaria in Kakuma refugee camp, Turkana, Kenya: facilitation of *Anopheles arabiensis* vector populations by installed water distribution and catchment systems. Malar J.

[11] Noor A, Gething P, Alegana V, Patil A, Hay S, Muchiri E, Juma E, Snow R (2009). The risks of malaria infection in Kenya in 2009. BMC Infect Dis.

[12] Mudhune S, Okiro E, Noor A, Zurovac D, Juma E, Ochola S, Snow R (2011). The clinical burden of malaria in Nairobi: a historical review and contemporary audit. Malar J.

[13] Okiro E, Alegana V, Noor A, Mutheu J, Juma E, Snow R (2009). Malaria paediatric hospitalization between 1999 and 2008 across Kenya. BMC Med.

[14] Ahn YJ, Shono T, Fukami J-I (1987). Effect of temperature on pyrethroid action to kdr-type house fly adults. Pestic Biochem Physiol.

[15] Miller TA, Adams ME, Coats JR (1982). Mode of action of pyrethroids. Insecticide Mode of Action.

[16] Hodjati MH, Curtis CF (1999). Effects of permethrin at different temperatures on pyrethroid-resistant and susceptible strains of *Anopheles*. Med Vet Entomol.

[17] Boina DR, Onagbola EO, Salyani M, Stelinski LL (2009). Influence of posttreatment temperature on the toxicity of insecticides against *Diaphorina citri* (Hemiptera: Psyllidae). J Econ Entomol.

[18] Sparks TC, Pavloff AM, Rose RL, Clower DF (1983). Temperature-toxicity relationships of pyrethroids on *Heliothis virescens* (F.) (Lepidoptera: Noctuidae) and *Anthonomus grandis grandis* Boheman (Coleoptera: Curculionidae). J Econ Entomol.

[19] Montgomery JC, Macdonald J (1990). Effects of temperature on nervous system: implications for behavioral performance. Am J Physiol.

[20] Gillooly JF, Brown JH, West GB, Savage VM, Charnov EL (2001). Effects of size and temperature on metabolic rate. Science.

[21] Hadaway AB, Barlow F (1963). The influence of environmental conditions on the contact toxicity of some insecticide deposits to adult mosquitos, *Anopheles stephensi* Liston. Bull Entomol Res.

[22] WHO (2006). Guidelines for testing mosquito adulticides for indoor residual spraying and treatment of mosquito nets.

[23] Okumu FO, Chipwaza B, Madumla EP, Mbeyela E, Lingamba G, Moore J, Ntamatungro AJ, Kavishe DR, Moore SJ (2012). Implications of bio-efficacy and persistence of insecticides when indoor residual spraying and long-lasting insecticide nets are combined for malaria prevention. Malar J.

[24] Etang J, Nwane P, Mbida JA, Piameu M, Manga B, Souop D, Awono-Ambene P (2011). Variations of insecticide residual bio-efficacy on different types of walls: results from a community-based trial in south Cameroon. Malar J.

[25] Glunt KD, Thomas MB, Read AF (2011). The effects of age, exposure history and malaria infection on the susceptibility of *Anopheles* mosquitoes to low concentrations of pyrethroid. PLoS ONE.

[26] Glunt KD (2013). Understanding the consequences of sub-lethal insecticide concentrations for insecticide resistance management and malaria control.

[27] R Development Core Team (2008). R: A language and environment for statistical computing.

[28] Cator LJ, Thomas S, Paaijmans KP, Ravishankaran S, Justin JA, Mathai MT, Read AF, Thomas MB, Eapen A (2013). Characterizing microclimate in urban malaria transmission settings: a case study from Chennai, India. Malar J.

[29] Lindblade KA, Dotson E, Hawley WA, Bayoh N, Williamson J, Mount D, Olang G, Vulule J, Slutsker L, Gimnig J (2005). Evaluation of long-lasting insecticidal nets after 2 years of household use. Trop Med Int Health.

[30] Hossain M, Curtis C (1989). Permethrin-impregnated bednets: behavioural and killing effects on mosquitoes. Med Vet Entomol.

[31] Balmert NJ, Rund SSC, Ghazi JP, Zhou P, Duffield GE (2014). Time-of-day specific changes in metabolic detoxification and insecticide resistance in the malaria mosquito *Anopheles gambiae*. J Insect Physiol.

[32] Murdock C, Paaijmans KP, Bell AS, King JG, Hillyer JF, Read AF, Thomas MB (2012). Complex effects of temperature on mosquito immune function. Proc Biol Sci.

[33] Li H, Feng T, Liang P, Shi X, Gao X, Jiang H (2006). Effect of temperature on toxicity of pyrethroids and endosulfan, activity of mitochondrial Na+ -K+ -ATPase and Ca2+ -Mg2+ -ATPase in *Chilo suppressalis* (Walker)(Lepidoptera: Pyralidae). Pestic Biochem Physiol.

[34] Ma Y-h, Gao Z-l, Dang Z-h, Li Y-f, Pan W-l (2012). Effect of temperature on the toxicity of several insecticides to *Apolygus lucorum* (Heteroptera: Miridae). J Pestic Sci.

[35] Musser FR, Shelton AM (2005). The influence of post-exposure temperature on the toxicity of insecticides to *Ostrinia nubilalis* (Lepidoptera: Crambidae). Pest Manag Sci.

[36] Royer TA, Elliott NC, Giles KL, Dean Kindler S (2011). Field efficacy of wintertime insecticide applications against greenbugs, *Schizaphis graminum* (Rondani) (Hemiptera: Aphididae) on winter wheat (*Triticumaestivum* L.). Crop Prot.

[37] Strode C, Donegan S, Garner P, Enayati AA, Hemingway J (2014). The impact of pyrethroid resistance on the efficacy of insecticide-treated bed nets against African anopheline mosquitoes: systematic review and meta-analysis. PLoS Med.

